# Anterior Cingulate Cortex Glutamate Levels and Sensory Integration Are Associated in Individuals With Social Anhedonia: A Comparison Between 3T and 7T MRS

**DOI:** 10.1002/pchj.70029

**Published:** 2025-06-29

**Authors:** Yun‐Ru Wang, Xuan Wang, Xin‐Lu Cai, Ling‐Ling Wang, Yu‐Xi Zhao, Li‐Xian Wang, Wei Mao, Zhu‐Jun Wei, Fangrong Zong, Yi Wang, Simon S. Y. Lui, Rong Xue, Raymond C. K. Chan

**Affiliations:** ^1^ Neuropsychology and Applied Cognitive Neuroscience Laboratory, State Key Laboratory of Cognitive Science and Mental Health Institute of Biophysics, Chinese Academy of Sciences Beijing China; ^2^ Department of Psychology The University of Chinese Academy of Sciences Beijing China; ^3^ Institute of Brain Science and Department of Physiology, School of Basic Medical Sciences Hangzhou Normal University Hangzhou China; ^4^ School of Psychology Shanghai Normal University Shanghai China; ^5^ Sino‐Danish College University of Chinese Academy of Sciences Beijing China; ^6^ State Key Laboratory of Cognitive Science and Mental Health Institute of Biophysics, Chinese Academy of Sciences Beijing China; ^7^ School of Artificial Intelligence Beijing University of Posts and Telecommunications Beijing China; ^8^ Department of Psychiatry, School of Clinical Medicine The University of Hong Kong Hong Kong Special Administrative Region China; ^9^ Beijing Institute for Brain Disorders Beijing China

**Keywords:** anhedonia, glutamate, magnetic resonance spectroscopy, neurological soft signs, schizotypy

## Abstract

Deficits in schizophrenia are linked to abnormalities in the glutamate (Glu) system, which are believed to result in neurological soft signs (NSS) and negative symptoms. This study investigated the relationship between Glu levels of the anterior cingulate cortex (ACC) and NSS, particularly sensory integration, in individuals with high and low levels of social anhedonia using 3 Tesla (T) and 7T Magnetic Resonance Spectroscopy (MRS). We recruited 16 participants with high social anhedonia and 18 with low social anhedonia and correlated their NSS scores with ACC Glu levels. While spectral quality metrics differed between field strengths, with 7T showing better spectral resolution and metabolite quantification reliability, both 3T and 7T data showed consistent correlation patterns. Our findings demonstrated that higher ACC Glu levels were associated with poorer sensory integration in high levels of social anhedonia across both field strengths, supported by both Pearson and Spearman rank correlations. Notably, the opposite pattern of association was found in people with low levels of social anhedonia at 7T. The Glu systems may be the common mechanisms for negative symptoms and NSS, highlighting its potential as a therapeutic target.

## 1 Introduction

Dysfunctions in the dopamine system are regarded as the core pathology of schizophrenia (Laruelle 2014), and antipsychotics mostly act on the dopamine system to alleviate positive symptoms (Howes et al. 2017; Miyamoto et al. 2012). Dopamine‐blocking antipsychotics are generally not effective for treating negative symptoms (Millan et al. 2014), which may involve other neurotransmitter abnormalities, such as the glutamatergic system (Correll and Schooler 2020; Galderisi et al. 2021; Poels et al. 2014). Besides positive and negative symptoms, schizophrenia patients also exhibit neurological soft signs (NSS) (Chan and Gottesman 2008; Chan et al. 2010, 2016; Zhao et al. 2014). NSS refers to subtle, non‐localizable impairments affecting sensory integration, motor coordination, and disinhibition (Chen et al. 1995; Heinrichs and Buchanan 1988). Evidence suggests that NSS may be a putative endophenotype for schizophrenia and is closely associated with negative symptoms (Chan and Gottesman 2008; Chan et al. 2016; Feng et al. 2020; Xu et al. 2016).

Interestingly, NSS and negative symptoms may originate from the same neural abnormalities. Previous behavioral studies strongly suggested the close association between NSS and negative symptoms (Bombin et al. 2005; Chan, Geng, et al. 2015; Chan, Shi, et al. 2015; de Bartolomeis et al. 2018; Fong et al. 2017; Iasevoli et al. 2018). Specifically, the severity of NSS is correlated with negative symptom severity, and such correlation is particular strong for sensory integration deficits and social withdrawal (Bombin et al. 2005). Moreover, NSS may mediate the effects of negative symptoms on functional outcomes (Fong et al. 2017). A recent functional magnetic resonance imaging (fMRI) study found that resting‐state functional connectivity between the cerebellum and the prefrontal cortex may underpin both NSS and negative symptoms (Cai et al. 2021). In ultra‐high‐risk populations, NSS predicted abnormal neural mechanisms and the development of negative symptoms (Mittal et al. 2014).

Anhedonia, defined as the diminished ability to experience pleasure, can be observed across psychiatric disorders and in subclinical populations (Liang et al. 2022; Chan et al. 2025; Wang et al. 2025). Within the schizophrenia spectrum, social anhedonia, defined as the reduced ability to experience pleasure from social interactions and relationships (Chapman et al. 1976), is a core negative symptom and the key feature of schizotypy (Chan et al. 2025; Meehl 1962). Importantly, social anhedonia is not exclusive to schizophrenia spectrum disorders but represents a dimensional symptom across a wide range of psychiatric conditions, including major depressive disorder, bipolar disorder; it can also manifest in mentally healthy individuals without clinical diagnoses (Blanchard et al. 2001; Pelizza and Ferrari 2009; Wang et al. 2025). In the schizophrenia spectrum, social anhedonia bears particular significance as it serves both as a negative symptom in diagnosed patients and as a schizotypal trait associated with psychosis proneness in non‐clinical populations (Chan et al. 2025; Wang et al. 2025). Recent factor analyses have indicated that social anhedonia and negative symptoms apparently load onto the same dimension (Cicero et al. 2019; Yang et al. 2022), suggesting shared underlying mechanisms.

Growing evidence supports the relationship between Glutamine (Glu) dysfunction, NSS, and negative symptoms. Schizophrenia patients have altered glutamatergic circuits involving the ACC, putamen, caudate, and thalamus (Walther and Strik 2012). These neural circuits are critically involved in both motor coordination and sensory processing, and their dysfunction contributes to both NSS manifestations and negative symptom expression. Previous studies have found associations between higher Glu levels at the ACC and more severe negative symptoms (Goldsmith and Rapaport 2020; Kruse and Bustillo 2022; Walther and Strik 2012). A recent study demonstrated a positive correlation between NSS and Glu levels at the ACC in schizophrenia patients, but such a correlation was not observed in healthy people (Cai et al. 2023). Interestingly, a disrupted Glu system is associated with impaired sensory integration, a domain of NSS. Using the combined magnetic resonance spectroscopy (MRS) and functional magnetic resonance imaging (fMRI) methods, Cai et al. (2023) reported that the Glu level at the ACC was correlated with functional activation and connectivity during a sensory integration task in schizophrenia patients. In people with low levels of social anhedonia, the gamma‐aminobutyric acid (GABA) level was positively correlated with sensory integration ability, indicating that higher GABA levels were associated with better performance (Cai et al. 2022).

The relationship between Glu and sensory integration appears to be complex. Higher Glu levels at the ACC have been associated with more severe negative symptoms in schizophrenia (Goldsmith and Rapaport 2020; Kruse and Bustillo 2022). Our previous studies suggested a differential relationship between neurotransmitter levels and sensory integration that may depend on baseline neural states. In individuals with low levels of social anhedonia, we found that GABA levels were correlated with sensory integration performance (Cai et al. 2022), while in patients with schizophrenia, Glu levels in the ACC were associated with altered functional activation during sensory integration tasks (Cai et al. 2023). These findings align with the Excitation‐Inhibition (E/I) imbalance model (Barch et al. 2001; Chun et al. 2018; Gao and Penzes 2015), which suggests that altered GABA levels can affect Glu function, potentially resulting in distinct functional outcomes in different clinical populations.

However, several limitations in previous research should be addressed. First, previous samples did not include medication‐naïve schizophrenia patients, although antipsychotics can significantly alter glutamatergic metabolite levels (Merritt et al. 2021). Second, investigations of the Glu system seldom recruited people with schizotypy, a subclinical condition along the schizophrenia spectrum (Johns and Van Os 2001; van Os et al. 2008). Schizotypy reflects a predisposition to schizophrenia and related personality traits, and is conceptualized as a dimensional construct (Meehl 1962; Raine 1991). Recent factor analyses indicated that social anhedonia and negative symptoms apparently loaded onto the same dimension (Cicero et al. 2019). Third, no previous study has directly measured Glu levels at the ACC in people with high levels of social anhedonia, nor investigated its relationship with sensory integration deficits. Our previous study only measured GABA levels, and reported its correlation with sensory integration in people with low (rather than high) levels of social anhedonia (Cai et al. 2023). Fourth, previous research mainly utilized 3T magnetic resonance imaging (MRI), which might have insufficient resolution to differentiate Glu from glutamine (Gln) signals (Grent‐'t‐Jong et al. 2022; Ramadan et al. 2013). Glx (combination of glutamate and glutamine) may be a crude measure with significant loss of signal data (Grent‐'t‐Jong et al. 2022; Ramadan et al. 2013; Sanaei Nezhad et al. 2018). On the contrary, 7T MRS offers distinct advantages through superior field strength, spectral resolution, and signal‐to‐noise ratios, enabling differentiation between Glu and Gln signals. Cramer‐Rao Lower Bounds (CRLBs) for Glu and Gln measurements at the ACC are significantly improved with 7T compared to 3T scanners (Pradhan et al. 2015), yet comparative studies across different MRS field strengths remain scarce.

Given these considerations, the present study aimed to examine the relationship between Glu levels at the ACC and sensory integration in people with high levels of social anhedonia, while directly comparing results obtained using 3T MRS and 7T MRS. Based on previous findings of the correlation of sensory integration with brain Glu levels (Cai et al. 2022; Cai et al. 2023), we hypothesized that (1) higher Glu levels at the ACC would correlate with more severe sensory integration deficits in people with high levels of social anhedonia, reflecting potential excitotoxicity in dysregulated neural circuits; and (2) lower Glu levels at the same region would correlate with more severe sensory integration deficits in people with low levels of social anhedonia, reflecting insufficient excitatory signaling in relatively healthy neural circuits. Additionally, we hypothesized that 7T MRS would provide more precise measurements and reveal stronger associations compared to 3T findings.

## 2 Methods

### 2.1 Participants

From 2021 to 2023, we recruited college students in Beijing, China, as participants. They completed the revised Chinese version of the Chapman Social Anhedonia Scale (CSAS; Chan et al. 2012; Chapman et al. 1976) via an online platform. Based on the CSAS cut‐off scores (Zhang et al. 2023), we selected 19 participants exhibiting high levels of social anhedonia (CSAS ≥ 20) and 19 participants showing low levels of social anhedonia (CSAS ≤ 12) for this study. The CSAS is a widely accepted tool for assessing social anhedonia (Cai et al. 2019; Chan, Geng, et al. 2015; Chan, Shi, et al. 2015; Wang et al. 2016).

The exclusion criteria included (1) any history of neurological disorders; (2) head trauma resulting in unconsciousness for over an hour; (3) any history of substance abuse; and (4) any contraindications for MRI scanning. To ensure the absence of psychiatric disorders, participants were assessed using the MINI‐International Neuropsychiatric Interview (Sheehan et al. 1998) by a trained experimenter. This study was conducted in accordance with the Helsinki Declaration and approved by the Ethics Committee of the Institute of Psychology, Chinese Academy of Science (Protocol number: H20022). Written informed consent was obtained.

### 2.2 Self‐Report Questionnaires and Behavioural Assessments

To evaluate social anhedonia, we utilized the Chinese version of the CSAS, which comprises 40 items and has demonstrated strong validity and reliability (Chan et al. 2012).

NSS were measured using the abridged version of the Cambridge Neurological Inventory (CNI), which includes three subscales: Motor Coordination (e.g., rapid finger tapping and pronation/supination), Sensory Integration (e.g., graphesthesia and stereognosis), and Disinhibition (e.g., saccade blink and wink) (Chan et al. 2009; Chen et al. 1995). Each item was rated on a dichotomized scale, where “0” indicated the absence and “1” for the presence of NSS, with higher scores reflecting greater severity. The three subscales of the CNI have shown high intra‐class correlation coefficients with the full scale (Chan et al. 2009).

### 2.3 MR Data Acquisition

MRI images were acquired using a SIEMENS 3T MR system (Siemens Prisma) and a SIEMENS whole‐body 7T MR research system (SIEMENS MAGNETOM) at the Institute of Biophysics, Chinese Academy of Sciences, Beijing, China. For the 3T MRI, a 20‐channel head coil was utilized, while a volume transmit, 32‐channel receive head coil (Nova Medial, MA, USA) was employed for the 7T MRI.

**FIGURE 1:**
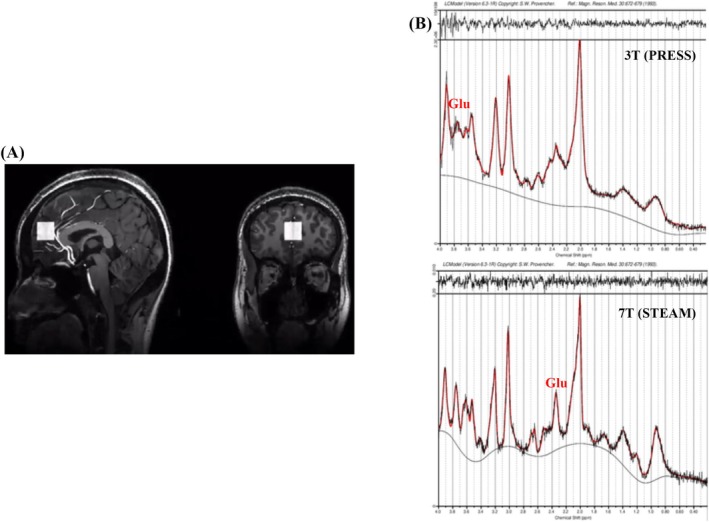
(A) The placement of voxel in the bilateral ACC. (B) LCModel fitting curves of 3T (PRESS) and 7T (STEAM) MRS.

For the 3T MR system, T1‐weighted structural imaging data were obtained using a 3D magnetization‐prepared rapid gradient‐echo (MPRAGE) sequence with the following parameters: repetition time (TR) = 2200 ms, echo time (TE) = 3.49 ms, inversion time (TI) = 1000 ms, Flip angle = 8°, field of view (FOV) = 256 mm × 224 mm, Matrix = 256 × 256, slice thickness = 1.00 mm, phase partial Fourier = 7/8. Following Modinos et al. (2017)'s study which measured glutamate levels at the ACC in individuals with high schizotypy, we collected our MRS data using a single‐voxel point‐resolved spectroscopy (PRESS) sequence (Modinos et al. 2017). The ^1^H‐MRS voxels (20 mm × 20 mm × 20 mm) were positioned in the bilateral ACC as the volume of interest (VOI) using the structural T1 scan as anatomical reference (Figure 1A). The parameters for this sequence were TR = 3000 ms, TE = 30 ms, number of averages = 128, bandwidth = 2000 Hz. VAPOR water suppression and automatic shimming of the instrument were performed using Siemens' built‐in advanced shim mode.

For 7T MR system, a 3D T1‐MPRAGE sequence was used with the following parameters: TR = 2600 ms, TE = 3.41 ms, Flip angle = 8°, FOV = 230 mm × 201 mm, Matrix = 384 × 384, slice thickness = 0.60 mm, acceleration factor = 2 (GRAPPA). ^1^H‐MRS data were acquired using an improved stimulated echo acquisition mode (STEAM) sequence to measure glutamate concentration in the brain. The parameters for this sequence were TR = 10,000 ms, TE = 5 ms, TM = 45 ms, Average = 32, voxel size = 20 mm × 20 mm × 20 mm, Number of averages = 32, Bandwidth = 4000 Hz. Water suppression was performed using VAPOR, and automatic shimming of the instrument was performed using Siemens' built‐in advanced shim mode. The scan protocol was consistent across all subjects.

### 2.4 ^1^H‐MRS Data Analysis

Water‐suppressed spectra were analyzed using LCModel version 6.3‐1 N. We adopted the commonly used criteria for spectral analysis to define poor‐quality data, and applied the following standards, that is, (1) Cramer‐Rao lower bounds (CRLBs) > 20% would be deemed poor fitting of the metabolite peak by the model; and (2) spectral values outside three standard deviations from the mean. The sample LCModel fitting curves comparing 3T (PRESS) and 7T (STEAM) MRS are shown in Figure 1B.

The reported metabolite levels from LCModel had to be corrected for voxel tissue composition due to the differential distribution of neurotransmitter metabolites in white matter (WM), gray matter (GM), and cerebrospinal fluid (CSF). The volume correction formula was as follows: Corrected metabolite level = Metabolite level × (WM proportion + 1.28 × GM proportion + 1.55 × CSF)/(WM proportion + GM proportion) (Bossong et al. 2019). Using the Osprey software (Oeltzschner et al. 2020), the header information of the MRS files was read to locate the regions of interest for structural imaging when acquiring spectroscopy data, segmenting the WM, GM, and CSF of each participant. These proportions obtained from the Osprey software were then input into the volume correction formula to obtain the corrected neurotransmitter levels. This work presented the glutamate levels, glutamine levels, and Glx levels, based on the levels corrected for volume.

Given that the 3T magnetic resonance instrument cannot effectively differentiate Glu from Gln, we focused on Glx as an approximate measure of Glu levels in the brain (Grent‐'t‐Jong et al. 2022; Ramadan et al. 2013; Sanaei Nezhad et al. 2018). The MRS sequence used at 7T could distinguish between the peaks of Glu and Gln, thus we focused on Glu levels (Pradhan et al. 2015). On the other hand, we reported the Glu, Gln, and Glx levels as references.

### 2.5 Statistical Analysis

Independent sample t‐tests were performed to investigate the differences between the two groups regarding demographic information, NSS, MRS quality parameters (FWHM, S/N, CRLBs), and the levels of Glu, Gln, and Glx. All statistical analyses were conducted using R software (R Core Team 2023).

To explore the relationship between the ACC Glu or Glx levels and NSS scores, Pearson correlation analyses were conducted within each group. Considering that age and gender can influence glutamatergic function (Brandt et al. 2016; Marsman et al. 2013; Merritt et al. 2021; Tayoshi et al. 2009), these variables were included as covariates in the correlation analyses between NSS scores and Glu or Glx levels. Additionally, as a subsidiary analysis, correlations for Gln levels were examined using the same methods.

Regarding sensory integration, given that the scores for high social anhedonia were exclusively 0 and 1, we additionally conducted Spearman rank correlation analyses to investigate the relationship between sensory integration scores and metabolite levels, specifically for the high levels of social anhedonia group.

In addition, to compare the data quality and metabolite measurements between 3T and 7T, *t*‐tests were performed to examine differences in spectral quality metrics (FWHM, SNR), metabolite levels (Glu, Gln, and Glx), and their corresponding CRLBs between the two field strengths. The significance level was set at *p* < 0.05 for all statistical tests.

## 3 Results

**FIGURE 2:**
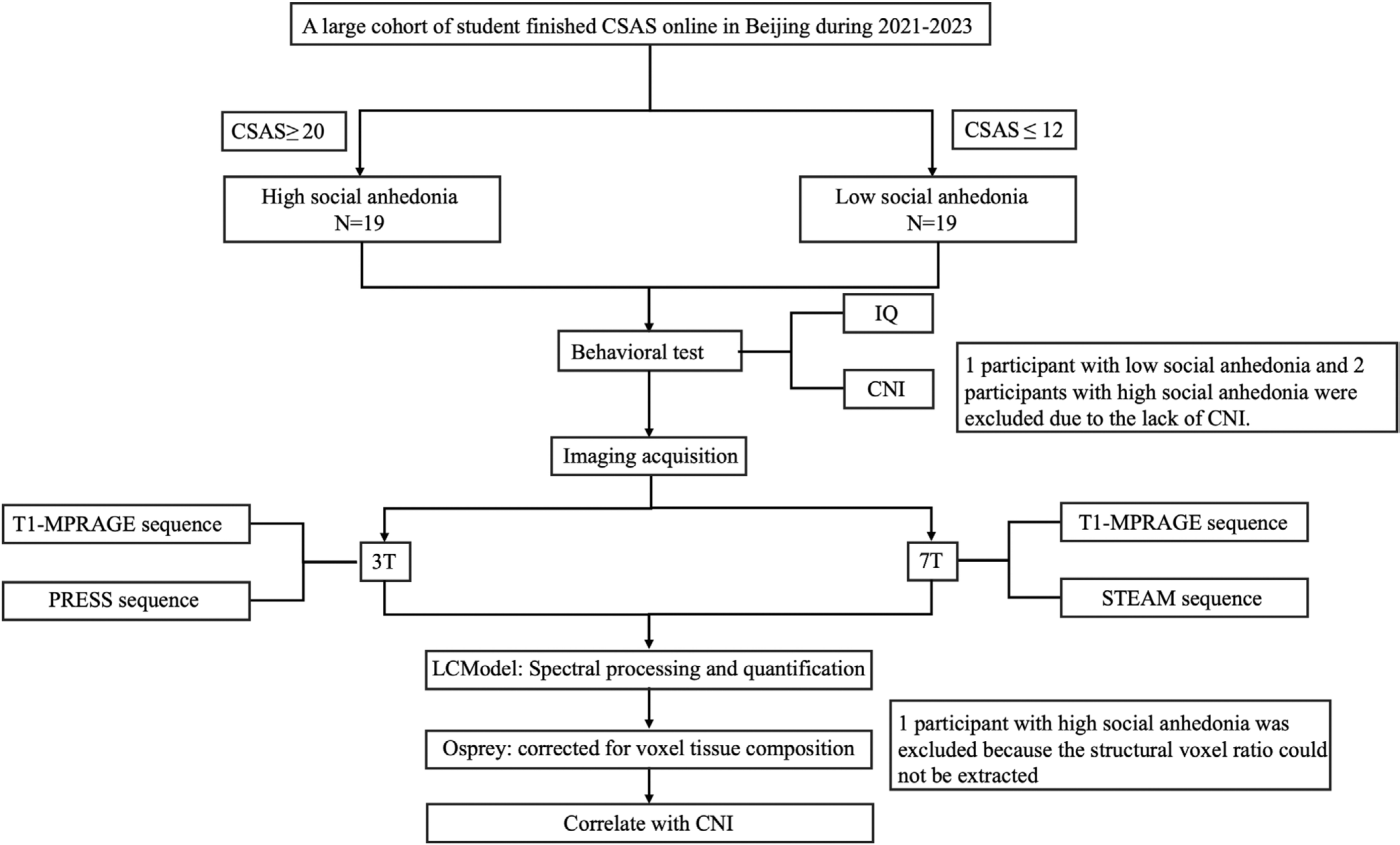
Flowchart of participant recruitment and data analysis.

We excluded one participant with low levels of social anhedonia and two participants with high levels of social anhedonia due to missing data of CNI, and another participant with high levels of social anhedonia because the structural voxel ratio could not be extracted (Figure 2). Our final sample comprised 16 and 18 participants with high‐ and low‐levels of social anhedonia respectively.

Table 1 presents the characteristics of participants with low (*N* = 18) and high (*N* = 16) levels of social anhedonia. The gender ratio did not differ between the two groups. However, those with high levels of social anhedonia were notably younger and had lower educational attainment compared to those with low levels of social anhedonia. Although participants with high levels of social anhedonia had higher scores in the NSS motor coordination, sensory integration, and disinhibition domains than participants with low levels of social anhedonia, the differences were not statistically significant.

**TABLE 1:** Demographic information and CNI scores for high and low social anhedonia.

	Low levels of social anhedonia (*N* = 18)		High levels of social anhedonia (*N* = 16)				
	Mean	SD	Mean	SD	*t*/*χ* ^2^	*p*	Cohen's *d*
Gender (male %)	33.33%		43.75%		0.07	0.787	0.05
Age (years)	22.61	2.23	19.81	1.60	4.24	**< 0.001**	1.43
Education (years)	16.94	2.48	13.88	1.86	4.11	**< 0.001**	1.39
CSAS	5.78	3.15	23.94	2.70	−18.10	**< 0.001**	−6.16
CNI							
Motor coordination	0.28	0.96	0.63	0.72	−1.20	0.238	−0.41
Sensory integration	0.22	0.55	0.31	0.48	−0.51	0.612	−0.17
Disinhibition	0.28	0.46	0.31	0.60	−0.19	0.853	−0.07
NSS total	0.78	1.40	1.25	1.06	−1.12	0.273	−0.38

*Note: p* < 0.05 are bold.

Abbreviations: CNI: Cambridge Neurological Inventory; CSAS: Chapman Social Anhedonia Scale; NSS: Neurological Soft Signs.

### 3.1 Glu or Glx Levels

In 3T MRS, the LCModel reported the mean SNR of 28.06 (SD = 9.45) and the mean CRLB of Glx (%) of 5.00 (SD = 1.28). As shown in Table 2, the SNR was higher in participants with low levels of social anhedonia than in participants with high levels of social anhedonia (*t* = 2.72, *p* = 0.011), indicating better data quality in the former group. The group difference in FWHM, CRLB, and structural voxel ratios all failed to reach statistical significance. The SNR was included as a covariate in the correlation analysis between Glu, Gln, and Glx levels and NSS.

**TABLE 2:** 3T and 7T MRS quality parameters and levels of glutamatergic neurotransmitters for high and low levels of social anhedonia.

	Low levels of social anhedonia (*N* = 18)		High levels of social anhedonia (*N* = 16)				
	Mean	SD	Mean	SD	*t*	*p*	Cohen's *d*
3T MRS quality parameters							
FWHM	0.06	0.01	0.06	0.03	−0.77	0.448	−0.27
SNR	31.78	10.30	23.88	6.39	2.72	**0.011**	0.91
CRLB of Glu	5.89	1.23	6.69	1.99	−1.39	0.178	−0.49
CRLB of Gln	9.61	3.29	9.19	1.47	0.49	0.626	0.16
CRLB of Glx	4.89	1.45	5.13	1.09	−0.54	0.593	−0.18
GM proportion	0.71	0.02	0.71	0.04	−0.22	0.831	−0.08
WM proportion	0.07	0.03	0.08	0.03	−0.12	0.902	−0.04
CSF proportion	0.22	0.04	0.21	0.03	0.19	0.853	0.06
Levels of Neurotransmitters in 3T							
Glu	10.50	1.95	9.45	2.44	1.38	0.180	0.48
Gln	7.63	1.50	8.01	1.35	−0.78	0.443	−0.27
Glx	18.13	2.99	17.46	3.19	0.63	0.533	0.22
7T MRS quality parameters							
FWHM	0.04	0.01	0.05	0.02	−2.05	0.053	−0.73
SNR	22.50	5.97	16.50	5.11	3.16	**0.003**	1.07
CRLB of Glu	2.67	0.69	3.31	0.79	−2.52	**0.017**	−0.87
CRLB of Gln	7.56	1.72	10.19	2.74	−3.31	**0.003**	−1.17
CRLB of Glx	2.72	0.67	3.50	0.82	−3.02	**0.005**	−1.05
GM proportion	0.79	0.06	0.79	0.04	−0.01	0.990	< 0.01
WM proportion	0.09	0.07	0.08	0.03	0.31	0.756	0.10
CSF proportion	0.12	0.03	0.13	0.03	−0.67	0.511	−0.23
Levels of Neurotransmitters in 7T							
Glu	9.14	1.00	8.91	1.13	0.61	0.544	0.21
Gln	2.60	0.34	2.52	0.39	0.61	0.545	0.21
Glx	11.73	1.11	11.43	1.43	0.69	0.497	0.24

*Note: p* < 0.05 are bold.

Abbreviations: CRLB: Cramer‐Rao lower bound; CSF: cerebrospinal fluid; FWHM: full width at half maximum; Gln: glutamine; Glu: glutamate; Glx: glutamate + glutamine; GM: gray matter; SNR: signal‐to‐noise ratio; WM: white matter.

In 7T MRS, the LCModel reported the mean SNR of 19.68 (SD = 6.28) and mean CRLB of Glu (%) of 2.97 (SD = 0.80). As shown in Table 2, the SNR was higher in participants with low levels of social anhedonia than in participants with high levels of social anhedonia (*t* = 3.16, *p* = 0.003). Moreover, the CRLBs for Glu, Gln, and Glx were significantly lower in participants with low levels of social anhedonia (*t* = −2.52, *p* = 0.017; *t* = −3.31, *p* = 0.003; *t* = −3.02, *p* = 0.005) than in participants with high levels of social anhedonia, indicating better data quality of 7T MRS in the former group. The SNR and CRLBs were therefore included as covariates in the correlation analysis between Glu, Gln, and Glx levels and NSS. However, FWHM and structural voxel ratios did not show significant differences between the two groups.

In the 3T and 7T data, differences between the two groups are not significant in Glu, Gln, or Glx levels in ACC (3T: *t* = 1.38, *p* = 0.180; *t* = −0.78, *p* = 0.443; *t* = 0.63, *p* = 0.533; 7 T: *t* = 0.61, *p* = 0.544; *t* = 0.61, *p* = 0.545; *t* = 0.69, *p* = 0.497) (see Table 2).

### 3.2 Relationship Between Glu Levels and NSS Scores

**FIGURE 3:**
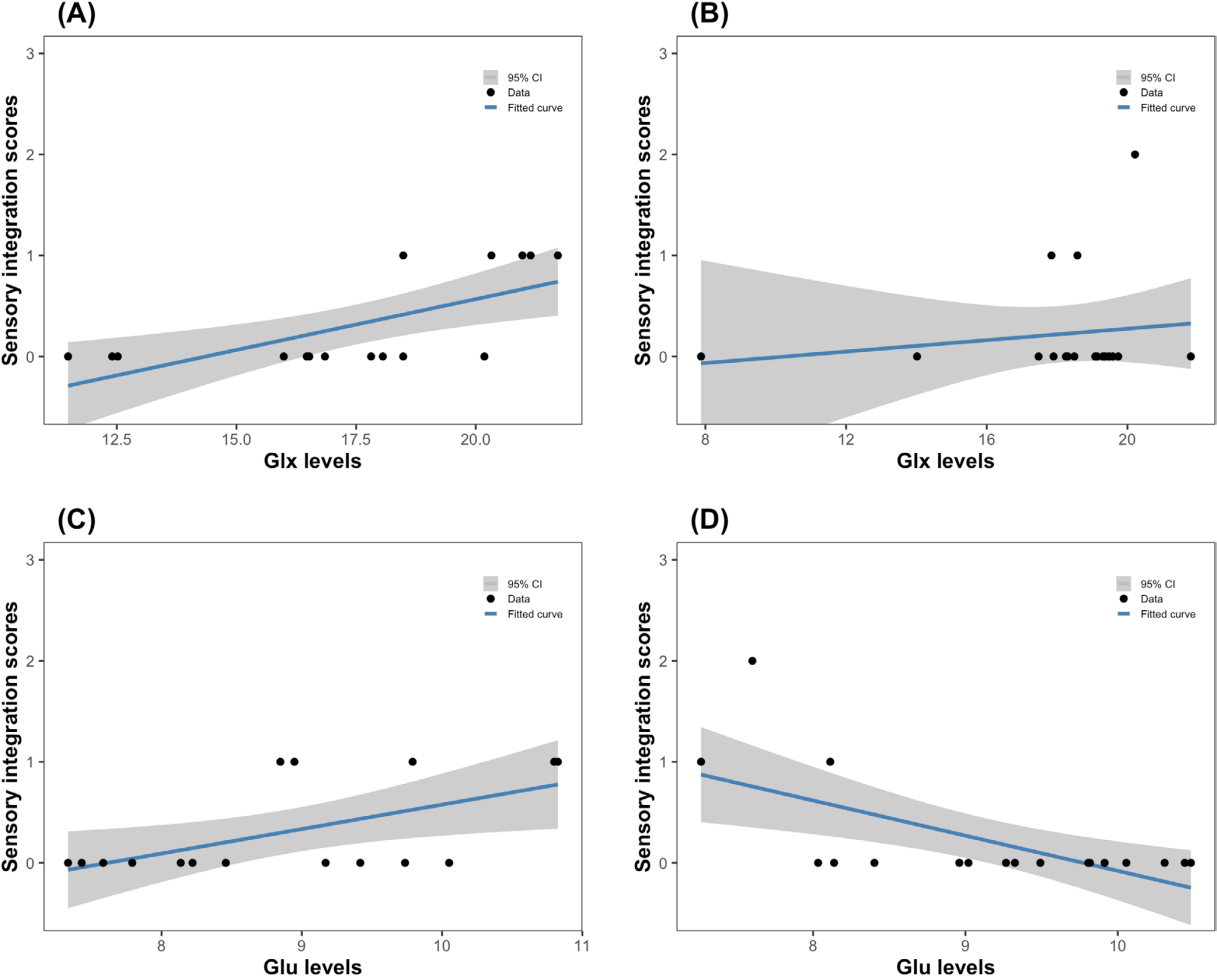
Pearson correlation analyses (without covariates) between metabolite levels and sensory integration scores. (A) In 3T MRS, scatterplot showing the correlation between Glx levels and sensory integration scores in participants with high levels of social anhedonia. (B) In 3T MRS, scatterplot showing the correlation between Glx levels and sensory integration scores in participants with low levels of social anhedonia. (C) In 7T MRS, scatterplot showing the correlation between Glu levels and sensory integration scores in participants with high levels of social anhedonia. (D) In 7T MRS, scatterplot showing the correlation between Glu levels and sensory integration scores in participants with low levels of social anhedonia.

For the 3T MRS data, we found a significant positive correlation between Glx levels and sensory integration scores in participants with high levels of social anhedonia (Figure 3A). This Pearson correlation persisted even after controlling for age, gender, and SNR (*r* = 0.668, *p* = 0.013) (Table 3). Conversely, in participants with low levels of social anhedonia, no correlation was found between Glx levels and sensory integration scores (Figure 3B) nor between other dimensions of NSS and Glx levels. These correlations remained non‐significant after controlling for age, gender, and SNR (Table 3). The Spearman rank correlation analysis further confirmed these relationships, showing significant correlations between sensory integration scores and Glu (*r* = 0.570, *p* = 0.021), Gln (*r* = 0.570, *p* = 0.021), and Glx levels (*r* = 0.775, *p* < 0.001) in participants with high levels of social anhedonia. However, it should be noted that no partial correlation was done for the Spearman rank correlation analysis.

**TABLE 3:** The partial correlation between CNI scores and Glu, Gln, Glx in 3T and 7T.

	Low levels of social anhedonia (*N* = 18)		High levels of social anhedonia (*N* = 16)	
	*r*	*p*	*r*	*p*
3T				
Motor coordination				
Glu	0.251	0.366	−0.098	0.750
Gln	0.184	0.512	0.076	0.806
Glx	0.268	0.335	−0.042	0.891
Sensory integration				
Glu	0.162	0.564	0.787	**0.001** ^a^
Gln	0.106	0.707	0.188	**0.539** ^a^
Glx	0.165	0.556	0.668	**0.013** ^a^
Disinhibition				
Glu	−0.184	0.512	0.009	0.976
Gln	−0.186	0.506	0.132	0.668
Glx	−0.225	0.421	0.061	0.842
NSS total				
Glu	0.184	0.511	0.249	0.411
Gln	0.112	0.692	0.204	0.504
Glx	0.183	0.514	0.272	0.369
7T				
Motor coordination				
Glu	−0.445	0.111	0.140	0.664
Gln	−0.658	**0.010**	0.025	0.939
Glx	−0.565	**0.035**	−0.017	0.958
Sensory integration				
Glu	−0.608	**0.021**	0.837	**< 0.001** ^b^
Gln	−0.663	**0.010**	0.700	**0.011** ^b^
Glx	−0.708	**0.005**	0.786	**0.002** ^b^
Disinhibition				
Glu	0.322	0.262	−0.236	0.460
Gln	0.436	0.119	−0.357	0.255
Glx	0.393	0.164	−0.422	0.172
NSS total				
Glu	−0.473	0.088	0.338	0.282
Gln	−0.606	**0.022**	0.160	0.620
Glx	−0.579	**0.030**	0.115	0.723

*Note:* For 3T data, the Pearson correlations were conducted while controlling for age, gender, and SNR. For 7T data, the Pearson correlations were conducted while controlling for age, gender, SNR, and corresponding CRLB values (i.e., Glu CRLB for Glu correlations, Gln CRLB for Gln correlations, and Glx CRLB for Glx correlations). *p* < 0.05 are bold.

Abbreviations: CNI: Cambridge Neurological Inventory; CRLB: Cramér‐Rao lower bounds; Gln: glutamine; Glu: glutamate; Glx: glutamate + glutamine; NSS: neurological soft signs; SNR = signal‐to‐noise ratio.

^a^ The Spearman rank correlation analysis further confirmed these relationships, showing significant correlations between sensory integration scores and Glu (*r* = 0.570, *p* = 0.021), Gln (*r* = 0.570, *p* = 0.021), and Glx levels (*r* = 0.775, *p* < 0.001) in participants with high levels of social anhedonia. However, it should be noted that no partial correlation was done for the Spearman rank correlation analysis.

^b^ The Spearman rank correlations confirmed these findings, showing significant correlations between sensory integration scores and Glu (*r* = 0.541, *p* = 0.030), Gln (*r* = 0.512, *p* = 0.043), and Glx levels (*r* = 0.541, *p* = 0.030) in participants with high levels of social anhedonia. However, it should be noted that no partial correlation was done for the Spearman rank correlation analysis.

For the 7T MRS data, we found significant positive correlations between Glu levels and sensory integration scores in participants with high levels of social anhedonia (Figure 3C). This Pearson correlation remained significant after adjusting for age, gender, SNR, and CRLB of Glu (*r* = 0.837, *p* < 0.001) (Table 3). Additionally, after controlling for age, gender, SNR, and CRLB, positive correlations were found between Gln and Glx levels with sensory integration scores (*r* = 0.700, *p* = 0.011; *r* = 0.786, *p* = 0.002). Among participants with low levels of social anhedonia, significant positive correlations were identified between Glu levels and sensory integration scores (Figure 3D). This Pearson correlation persisted after controlling for age, gender, SNR, and CRLB (*r* = −0.608, *p* = 0.021) (Table 3). Furthermore, after controlling for age, gender, SNR, and CRLB, Gln levels were negatively correlated with motor coordination scores, sensory integration scores, and NSS total scores (*r* = −0.658, *p* = 0.010; *r* = −0.663, *p* = 0.010; *r* = −0.606, *p* = 0.022), and Glx levels were negatively correlated with motor coordination scores, sensory integration scores, and NSS total scores (*r* = −0.565, *p* = 0.035; *r* = −0.663, *p* = 0.010; *r* = −0.579, *p* = 0.030). The Spearman rank correlations confirmed these findings, showing significant correlations between sensory integration scores and Glu (*r* = 0.541, *p* = 0.030), Gln (*r* = 0.512, *p* = 0.043), and Glx levels (*r* = 0.541, *p* = 0.030) in participants with high levels of social anhedonia. However, it should be noted that no partial correlation was done for Spearman rank correlation analysis.

### 3.3 Comparison Between 3T and 7T Measurements

**FIGURE 4:**
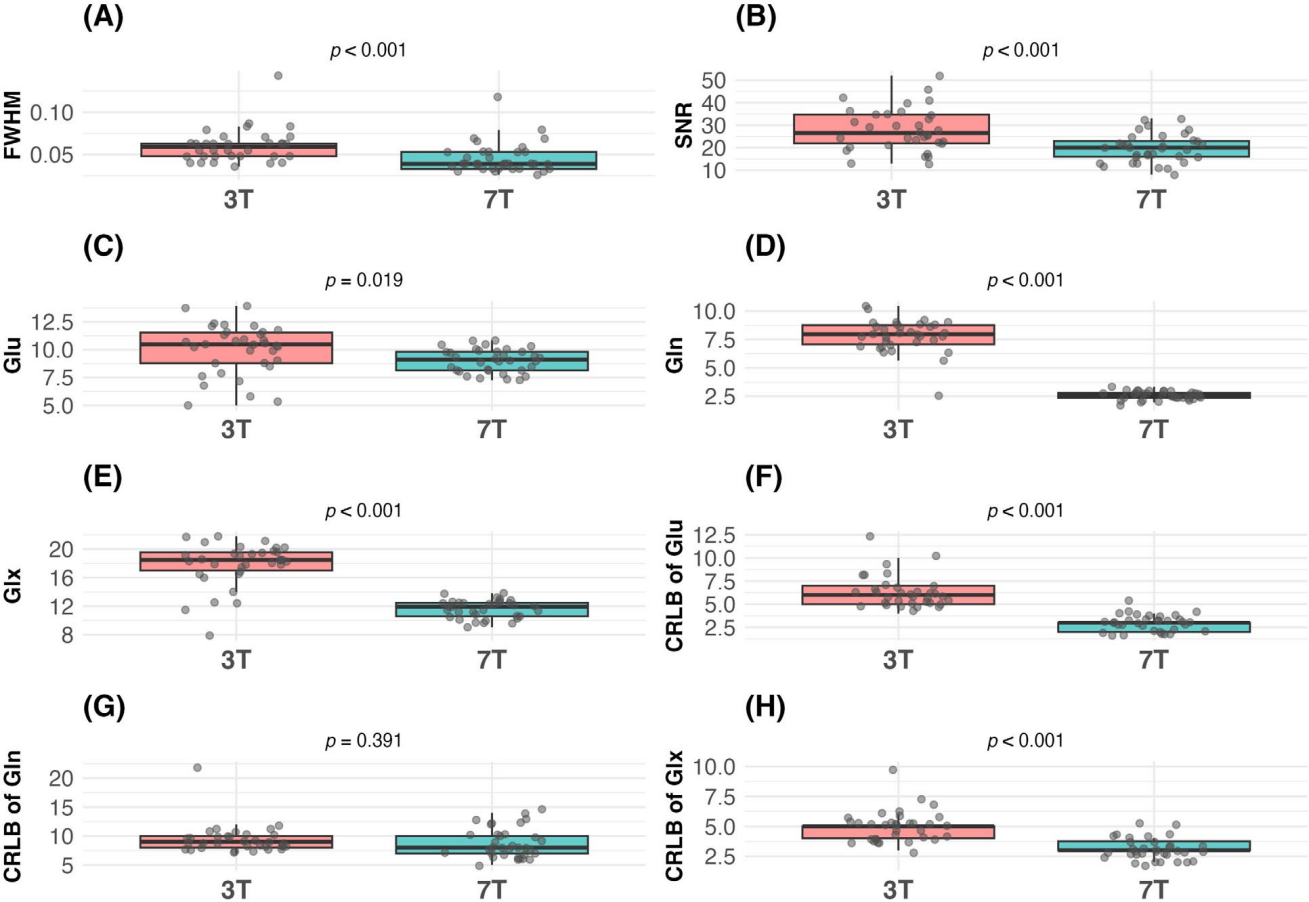
Comparison of MRS quality metrics and metabolite levels between 3T and 7T field strengths. (A) FWHM values showing improved spectral resolution at 7T. (B) SNR comparison between field strengths. (C–E) Metabolite levels including Glu, Gln, and Glx. (F–H) CRLBs for Glu, Gln, and Glx, demonstrating enhanced quantification reliability at 7T for Glu and Glx measurements.

Analysis of spectral quality metrics between field strengths revealed that FWHM was significantly lower in 7T MRS (Figure 4A; *t* = 5.01, *p* < 0.001), indicating enhanced spectral resolution. While SNR was higher in 3T MRS (Figure 4B; *t* = 4.38, *p* < 0.001), metabolite measurements showed that Glu (Figure 4C; *t* = 2.46, *p* = 0.019), Gln (Figure 4D; *t* = 20.99, *p* < 0.001), and Glx levels (Figure 4E; *t* = 11.27, *p* < 0.001) were lower in 7T MRS. Notably, CRLBs improved in 7T MRS for both Glu (Figure 4F; *t* = 10.77, *p* < 0.001) and Glx (Figure 4H; *t* = 6.88, *p* < 0.001), though Gln CRLBs showed no significant difference (Figure 4G; *t* = 0.87, *p* = 0.391).

## 4 Discussion

To our knowledge, this study was one of the first few investigations on the relationship between Glu and sensory integration in people with high and low levels of social anhedonia. We also directly compared the Glu levels as measured by using 3T and 7T MRS. Our findings can be summarized as follows. First, the sensory integration domain of NSS in people with high levels of social anhedonia was positively correlated with the Glu levels at ACC, as measured by using 3T and 7T MRS. These correlations remained significant in Spearman rank correlation analyses, supporting the robustness of our findings across different statistical approaches. Second, the sensory integration domain of NSS in people with low levels of social anhedonia was negatively correlated with the Glu levels at ACC, as measured by using 7T rather than 3T MRS. Third, the magnitude (correlation coefficients) of such correlation appeared to be larger when a 7T scanner was used. Fourth, comparison between 3T and 7T measurements revealed that, while SNR was higher in 3T MRS, 7T MRS demonstrated superior spectral resolution with lower FWHM values and improved metabolite quantification reliability as indicated by lower CRLBs for Glu and Glx. Together, our findings suggested opposite patterns of correlations of the NSS sensory integration domain scores with Glu levels at ACC in people with high versus low levels of social anhedonia, with 7T MRS providing more precise metabolite measurements despite certain technical trade‐offs.

In people with high levels of social anhedonia, elevated Glu levels in the ACC are associated with poorer sensory integration, which in turn correlates with more severe NSS and social anhedonia. By contrast, people with low levels of social anhedonia exhibit increased Glu levels at ACC, which are correlated with better sensory integration, suggesting a potential adaptive role of glutamatergic modulation in this group (Cai et al. 2022). The opposite patterns highlight the differential involvement of Glu metabolism in sensory processing across the spectrum of social anhedonia (Wang et al. 2024). Similarly, previous studies have found that Glu levels were negatively correlated with the performance of cognitive theory of mind in people with high levels of social anhedonia but not their counterparts without social anhedonia (Chen et al. 2022). According to the work of Wang et al. (2024), the E/I balance was negatively correlated with social cognition in people with high levels of social anhedonia. In contrast, no such correlation was observed in people without social anhedonia. Cognitive theory of mind and social cognition involve sensation and perception, indicating the difference between the two groups may be related to sensory integration. The Glu system may serve as a common mechanism underlying negative symptoms and NSS. However, our findings align with prior evidence that Glu dysfunction in the ACC is implicated in both NSS and negative‐symptom‐like traits (social anhedonia) in subclinical populations.

The results of this study were consistent with our first hypothesis, and concurred with previous findings. Our previous studies reported that impaired sensory integration was positively correlated with ACC Glu levels in patients with schizophrenia. We also observed a similar phenomenon in our subclinical sample, that is, higher ACC Glu levels in people with schizophrenia spectrum disorders were associated with more robust brain activity signals/functional connectivity (Cai et al. 2023). Our research findings were consistent with several other studies. NSS may be manifestations of abnormalities in perceptual function (Chen et al. 1995; Heinrichs and Buchanan 1988). Previous research has revealed that abnormalities in the Glu system might further lead to deficits in perceptual basic processing (Javitt 2015; Javitt and Freedman 2015; Rowland et al. 2016; Strube et al. 2020; Umbricht et al. 2002). For example, animal studies have found that injection of N‐methyl‐D‐aspartate Glu receptor (NMDAR) antagonists could impair rats' multi‐channel sensory integration in behavioral experiments (Cloke et al. 2016; Cloke and Winters 2015; Jacklin et al. 2012). Research on neuropharmacology showed that healthy subjects who were injected with NMDAR antagonists exhibited deficits in sensory information processing, similar to schizophrenia patients (Strube et al. 2020). Together, prior results suggested that abnormal Glu levels in the brain may ultimately manifest as deficits in sensory processing. Moreover, abnormalities in the Glu system in schizophrenia patients can lead to deficits in visual processing (Javitt 2015; Javitt and Freedman 2015).

Consistent with our second hypothesis, 7T MRS demonstrated several advantages over 3T scanning, though with some important nuances. Previous research has indicated that higher field strengths can improve sensitivity and measurement precision for key metabolites like Glu, Gln, and aspartate (Ladd et al. 2018; McCarthy et al. 2022). While our findings showed unexpectedly higher SNR in 3T compared to 7T MRS, likely due to the different pulse sequences employed (PRESS vs. STEAM), 7T MRS exhibited superior performance in other crucial metrics. Specifically, 7T MRS demonstrated better spectral resolution with lower FWHM values and more reliable metabolite quantification as evidenced by lower CRLBs for both Glu and Glx. These findings aligned with previous results, and suggested that higher field strengths could enable better differentiation between Glu and Gln signals (McCarthy et al. 2022). The improved precision in metabolite quantification (but lower SNR) using 7T MRS concurred with the earlier work (Pradhan et al. 2015) that higher field strengths could enhance separation of closely spaced resonances. The technical advantages of 7T MRS are crucial for psychiatric research, because accurate quantification of specific neurotransmitters is necessary. Similar to previous literature supporting the benefits of ultra‐high‐field MRS (Ladd et al. 2018), our findings indicated that 7T MRS is preferred for precise differentiation of specific metabolites to clarify neural mechanisms.

Our study has several limitations. First, we only measured Glu levels during the resting state. With the latest advancements in MR technology, functional magnetic resonance spectroscopy (^1^H fMRS) can directly measure neural activity related to behavior, and its results are much more reliable (Stanley and Raz 2018). Future research could consider employing ^1^H fMRS for further exploration. Second, our sample size was small, limiting our analysis on the binary scores (0 or 1) of sensory integration in the high social anhedonia group. Although Spearman rank correlations were used to partly address this problem, a larger sample size would be necessary to validate our findings. Moreover, we did not include clinical groups or people with ultra‐high risk (Chan et al. 2018). A better approach should recruit a larger sample with more diverse populations such as schizophrenia patients and ultra‐high‐risk groups to test the replicability of our results. Third, the use of different pulse sequences between 3T (PRESS) and 7T (STEAM) MRS acquisitions may have confounded the results of direct comparisons of certain quality metrics, particularly affecting SNR measurements and making it challenging to fully demonstrate the technical advantages of 7T MRS. Fourth, our study only focused on the relationship between Glu and sensory integration. Previous research suggested that GABA signals and the E/I balance were involved in sensory integration function (Barch et al. 2001; Chun et al. 2018; Gao and Penzes 2015). Future research should study the relationships between neurotransmitters like GABA and sensory integration, to enrich our understanding of the relationship between the E/I balance and sensory integration. Lastly, while social anhedonia is a core feature of the schizophrenia spectrum, it can also be observed in other psychiatric conditions such as depression and anxiety disorders. However, our study did not assess depressive or anxiety symptoms, which may confound the interpretation of the relationship of social anhedonia with glutamatergic function. Future research should include standardized measures (e.g., the Beck Depression Inventory, the State‐Trait Anxiety Inventory) covering both clinical symptoms, and to disentangle symptom‐specific versus transdiagnostic neural correlates.

## 5 Conclusions

We demonstrated differential associations between ACC Glu levels and sensory integration among people with high versus low levels of social anhedonia. Elevated Glu levels were linked to impaired sensory integration in people with high levels of social anhedonia, while the opposite pattern of association was observed in people with low levels of social anhedonia. The 7T MRS yielded more robust results than the 3T MRS. Our findings underscored the involvement of the Glu system in sensory integration deficits and negative‐symptom‐like behavioral manifestations in people with social anhedonia, implicating its potential as a therapeutic target. Further research is warranted to elucidate the underlying mechanisms and replicate our preliminary findings using larger and more diverse samples.

## Disclosure

The funding agents had no further role in the study design; in the collection, analysis and interpretation of the data; in the writing of the manuscript; and in the decision to submit the paper for publication.

## Conflicts of Interest

The authors declare no conflicts of interest.

## Data Availability

Research data are not shared.
